# Zinc Finger E-Box Binding Homeobox Family: Non-Coding RNA and Epigenetic Regulation in Gliomas

**DOI:** 10.3390/biomedicines11051364

**Published:** 2023-05-05

**Authors:** Bartosz Lenda, Marta Żebrowska-Nawrocka, Grzegorz Turek, Ewa Balcerczak

**Affiliations:** 1 Laboratory of Molecular Diagnostics, Department of Pharmaceutical Biochemistry and Molecular Diagnostics, BRaIN Laboratories, Medical University of Lodz, Czechoslowacka 4, 92-216 Lodz, Poland; marta.zebrowska@umed.lodz.pl (M.Ż.-N.); ewa.balcerczak@umed.lodz.pl (E.B.); 2 Department of Neurosurgery, Bródnowski Masovian Hospital, Kondratowicza 8, 03-242 Warsaw, Poland; turek.grz@gmail.com

**Keywords:** glioma, glioblastoma, EMT, ZEB, ncRNA, miRNA, lncRNA, epigenetics

## Abstract

Gliomas are the most common malignant brain tumours. Among them, glioblastoma (GBM) is a grade four tumour with a median survival of approximately 15 months and still limited treatment options. Although a classical epithelial to mesenchymal transition (EMT) is not the case in glioma due to its non-epithelial origin, the EMT-like processes may contribute largely to the aggressive and highly infiltrative nature of these tumours, thus promoting invasive phenotype and intracranial metastasis. To date, many well-known EMT transcription factors (EMT-TFs) have been described with clear, biological functions in glioma progression. Among them, EMT-related families of molecules such as SNAI, TWIST and ZEB are widely cited, well-established oncogenes considering both epithelial and non-epithelial tumours. In this review, we aimed to summarise the current knowledge with a regard to functional experiments considering the impact of miRNA and lncRNA as well as other epigenetic modifications, with a main focus on ZEB1 and ZEB2 in gliomas. Although we explored various molecular interactions and pathophysiological processes, such as cancer stem cell phenotype, hypoxia-induced EMT, tumour microenvironment and TMZ-resistant tumour cells, there is still a pressing need to elucidate the molecular mechanisms by which EMT-TFs are regulated in gliomas, which will enable researchers to uncover novel therapeutic targets as well as improve patients’ diagnosis and prognostication.

## 1. Introduction

Gliomas are the most common primary malignant brain tumours. Possibly, they originate from neural stem cells and are typically characterised by diffuse and extensive growth. The most common and most malignant type of glioma (grade four, IDH1/2 wt according to World Health Organisation) is glioblastoma (GBM) [1,2,3]. The median survival of patients with GBMs amounts to approximately 15 months when standard available treatment is used. This includes a combination of surgical resection/biopsy and adjuvant treatment such as radiotherapy and chemotherapy. Despite significant advances in the available treatment options and the emergence of several novel therapeutic tools, GBM still remains a deadly disease with a poor prognosis [4,5,6]. This may be due to the complex molecular heterogeneity which confers varied molecular and clinical features on individual tumours. That said, over the years different types of glioma classification have been proposed, including circumscribed gliomas/diffuse gliomas, WHO grades, primary/secondary tumours, adult/paediatric gliomas, as well as transcriptional glioma subtypes (proneural, neural, classical and mesenchymal). Considering genetic criteria, gliomas can be divided into gliomas with IDH1/2 gene mutations and gliomas with IDH1/2 wild type genes. Although these two groups share many similarities in terms of histological characteristics, they are very distinct regarding the molecular cascades which drive their progression; hence, they impact patients’ outcomes differentially. Other genetic markers including mutations in EGFR, PDGFRA and NF1 genes have been proposed as well, with a varied impact on tumourigenesis and patient outcomes. Therefore, novel therapeutic targets in the treatment of gliomas should focus on the molecular mechanisms underlying their aggressive behaviour and metastasis [7,8].

Epithelial to mesenchymal transition (EMT) is a complex, molecular program that plays an essential part in the progression of epithelial tumours to invasive phenotypes [9]. However, gliomas do not engage in a typical EMT pathway, as these tumours do not originate from classical epithelial tissue [10]. Nevertheless, EMT-like changes, which are the main cause of increased invasiveness, stem cell signature and loss of cell–cell contact, can contribute extensively to increased progression and metastasis also in non-epithelial tumours, including gliomas. Accordingly, EMT-promoting transcription factors (EMT-TFs), such as Snail (SNAI1), Slug (SNAI2), Twist1 and Twist2 appear to play pivotal roles in various aspects of tumourigenic processes [11,12]. Among them, ZEB family members, such as ZEB1 and ZEB2, i.e., zinc finger E-box binding homeobox proteins, are also important modulators of the molecular network in gliomas with a substantial impact on the proliferation, invasion and migration of tumourigenic cells [13,14,15,16,17,18]. As key canonical EMT transcription factors, they repress downstream regulators of an epithelial phenotype while activating those which increase mesenchymal features [19]. To date, ZEBs were also indicated in the development and progression of many other types of human cancer, such as prostate cancer, breast cancer and colorectal cancer [20,21,22,23]. Multiple studies have shown that the impact of ZEBs on tumour progression is associated with different regulatory mechanisms, such as post-translational modifications, the action of other EMT-related molecules and some non-coding RNAs [24]. In this paper, we decided to review ZEB-impacting micro- and long non-coding RNAs with an exclusive focus on GBMs and other gliomas.

## 2. MiRNA Targeting ZEBs

MicroRNAs (miRNAs) are a group of endogenous, non-coding, 21–25 nucleotide-long RNAs. As important gene expression regulators, miRNAs are commonly known to base pair with the 3′ untranslated regions (3′-UTRs) of their downstream mRNA targets, thus driving the posttranscriptional inhibition [25,26]. Depending on the molecular context, miRNAs can exert activating or inhibitory effects on various tumourigenic processes, such as proliferation, migration, invasion and apoptosis [27,28,29,30]. To date, many miRNAs have been identified to affect glioma progression both as oncogenes (oncomirs) and tumour suppressors, including miR-139-5p [31], miR-595 [32], miR-296-5p [33] and miR-429 [34], to mention only a few. As miRNAs are potent regulators of glioma development, it is clear that they constitute a potential tumour therapeutic target.

The miRNAs that are known to affect ZEB1/ZEB2 act as tumour suppressors and are significantly downregulated in various glioma samples and cell lines. Various biological functions of ZEB miRNAs have been described (Table 1). Many miRNAs are believed to play an important role in EMT-associated events, as they target ZEBs directly or by their upstream regulators and affect other EMT-related factors. For example, miR-590-3p blocks EMT and metastasis by targeting ZEB1 and ZEB2 in GBM cell lines [35]; the miR-940/ZEB2 axis constitutes an important regulator of glioma cell aggressive phenotype, which was additionally proven in an orthotopic GBM mouse model [36]; and miR-205 suppresses the oncogenic potential of ZEB1 via the Akt/mTOR signalling pathway, which inhibits motile phenotype and EMT in GBM cells [37]. MiR-200a exerts an inhibitory effect on ZEB1 in glioma cells, as noted in a study exploring the role of glioma-related EMT in reference to the Tissue factor (TF) [38].

### 2.1. MiR-200 Family

It is widely known that the interplay between ZEBs and miR-200 family members constitutes a double negative feedback loop and largely affects EMT-related signalling pathways [52]. The miR-200 family (miR-200s) is a tumour-suppressive group of miRNAs which consists of miR-200c/miR-141 and miR-200a/miR-200b/miR-429 clusters [53]. According to reports, miR-200s can affect ZEB1 and/or ZEB2 expression in many types of cancer, such as gastric cancer [54], non-small lung cancer [55] and oral squamous cell carcinoma [56]. In gliomas, miR-200c as well as miR-141 can inhibit glioma cell growth and migration by targeting ZEB1 [42].

Notably, the interaction between miR-200c, ZEB1 and the epidermal growth factor receptor amplification pattern seems to be particularly interesting. Epidermal growth factor receptor (EGFR) amplification is observed in about 35–70% of GBM cases [43]. As different GBM samples are highly varied in terms of EGFR alteration type, three groups of EGFR amplification status can be distinguished. They consist of GBMs with double minutes (high EGFR amplification level), GBMs with insertions on chromosome 7 (low EGFR amplification level) and GBMs with no EGFR amplification [44]. Different EGFR gene and protein alterations have been proposed as potential prognostic indicators or therapy response predictors—that said, different types of aberrant EGFR expression patterns could be used to asses individual schedules of treatment in cancer patients. However, the clinical value of EGFR is not clear yet and requires further research [8,57]. Serna et al. [44] reported that miR-200c expression level differs between primary GBM tumour samples with and without EGFR amplification, and that miR-200c and E-cadherin are downregulated in the high-level EGFR amplification group. In turn, tumours without EGFR amplification showed significantly lower ZEB1 expression [44]. Considering the subset of migration-related miRNAs, miR-200c has the most varied expression in a group of GBM patients regarding EGFR amplification status [43]. Subsequent experiments conducted with three patient-derived GBM cultures, which were varied in terms of EGFR amplification status, additionally elucidated the impact of EGFR amplification on miR-200c/ZEB1 interaction; although miR-200c overexpression exerts an inhibitory effect on ZEB1 regardless of EGFR amplification status, the expression level of ZEB1 was upregulated by miR-200c inhibition only in non-EGFR-amplifying cells, which according to authors may suggest the existence of some additional mechanism affecting ZEB1 in the context of an EGFR amplification environment [43]. As the EGF/EGFR pathway has a broad impact on various cellular processes, it states an interesting research direction also in the context of other EMT-related TFs and ncRNAs. They include not only alterations in its amplification patterns but also other EGFR gene aberrations, such as the mutational variant EGFRvIII, commonly associated with aggressive GBM cell behaviour [57].

In addition to ZEB-related double negative feedback loop and EGFR amplification status, there are also many other epigenetic mechanisms that can affect miR-200s in tumour progression [58,59,60]. One of the epigenetic regulators known as an important factor in various neoplasms (e.g., prostate cancer, colorectal cancer, breast cancer) is methyl CpG-binding protein 2 (MeCP2) [61,62,63], which belongs to the methyl CpG binding domain (MBD) family. In LN-25 and U251 glioma cell lines, knockdown of MeCP2 resulted in decreased expression of the EMT-related markers, i.e., ZEB1, ZEB2 and Twist1; a similar effect was also observed in a xenograft mouse model [45]. In addition, MeCP2 inhibited miR-200c expression in glioma cells and was negatively correlated with miR-200c expression in glioma tissues. As a mechanism, epigenetic repression caused by MeCP2 and the suppressor of variegation 39H1 (SUV39H1), resulting in miR-200c promoter repression, has been proposed [45]. As the MeCP2 has been reported in several “MeCP2-related disorders” and is considered “an essential reader of DNA methylation in the brain”, it remains a good point of interest in further research of molecular interactions in glioma pathogenesis and other CNS-related diseases [64].

### 2.2. Cancer Stem Cell

The ability to self-renew, multi-potent differentiation as well as intrinsic tumourigenic potential are some of the key characteristics attributed to cancer stem cells (CSCs). It is well-known that GBM harbours a population of CSCs that are commonly known as glioblastoma stemlike cells (GSCs). GSCs largely contribute to tumour recurrence and constitute a valuable target for GBM treatment [65,66]. As CSCs are at the top of the ontogenic hierarchy not only in normal organs but also in tumours, they have been considered a valuable target for cancer therapy and recurrence for many years. That said, various strategies have been proposed in order to effectively combat GSCs, including targeting genetic and epigenetic regulation, a tumour’s microenvironment and immunotherapy-based approaches [67]. For example, valproic acid (histone deacetylase inhibitor) can exert an inhibitory effect on GSCs proliferation and motility because it induces DNA methylation changes in the Wnt/β-catenin signalling pathway and inhibits the expression of key EMT markers—Snail and Twist1 [68].

Another transcription factor which constitutes a hallmark signature of the occurrence and development of cancer is MYC. As a key epigenetic regulator, it induces stem cell phenotype, inhibits cellular senescence and blocks the differentiation of tumour cells. Thus, MYC represents a valuable target for molecular treatment in various types of human neoplasms [69,70]. Considering GSCs, MYC has been shown to possess a broad impact on a stem-related transcriptome by interacting with other transcription factors in promoter regions of many downstream genes [40]. Several studies reported that MYC inhibition can be effective in cancer treatment, due to the action of a 90-aa polypeptide Omomyc [71,72]. Accordingly, Galardi et al. [40] showed that in patient-derived GSC lines, Omomyc blocks the activity of key proteins with CSC phenotype and affects various miRNAs associated with tumour progression. For example, Omomyc increased the expression of the ZEB1-suppressing miR-200a/miR-200b/miR-429 cluster, which is well-known due to its anti-proliferative activity in cancer cells.

Notably, aggressive infiltration to adjacent tissue and increased motility of cancer cells is largely dependent on the proteolytic activity of numerous matrix metalloproteinases (MMPs), which are closely linked to EMT-associated tumour progression and can be released by tumourigenic cells themselves. MMPs are a crucial component of the tumour microenvironment in regulating angiogenesis and disseminating metastatic lesions outside the tumour zone [73]. One of the members of the A Disintegrin and Metalloproteinase (ADAM) family of zinc-dependent proteinases—ADAMDEC1 (which is secreted by GSCs)—is known to release Fibroblast growth factor 2 (FGF2) from the extracellular matrix (ECM) that stimulates FGFR1 activity. FGFR1 via ERK1/2 upregulates ZEB1, which rescues ADAMDEC1 expression from the inhibitory effect of miR-203. Hence, the pathway constitutes a positive feedback loop in the maintenance of stemness in the GSC population [48].

### 2.3. Predicting Prognosis

Many miRNAs are indicated as potential biomarkers in cancer, and the fact that they can be utilised in terms of their diagnostic and/or prognostic value is still extensively studied [39,74,75,76,77]. Among the ZEB family-related miRNAs, there are also a few examples. Downregulation of the ZEB2 suppressor miR-622 correlated with advanced WHO pathological grade and low Karnofsky performance score (KPS) in 108 tissue samples obtained from glioma patients (Pearsons’s Chi-square test). Notably, glioma specimens were varied in terms of WHO grades, comprising grade I–IV tumours (including 30 patients with GBMs) and receiving no treatment prior to tumour resection [49]. As for survival analysis, Kaplan–Meier curves also indicated a significant association between the downregulation of miR-622 expression and a low overall survival rate. To conclude, miR-622 downregulation, advanced WHO tumour grade and low KPS could be utilised as independent poor prognostic factors, as was indicated by Cox regression analysis [49]. Likewise, miR-769-3p is another ZEB2-suppressing molecule with potential prognostic value. Wang et al. [50] showed that miR-769-3p expression was significantly associated with the WHO grade and KPS both in tumour tissues and serum samples obtained from 113 primary glioma patients (58 patients with low grade gliomas and 55 patients with high grade gliomas) with no prior treatment (Chi-square test). Kaplan–Meier analysis revealed a close correlation between low miR-769-3p tumour expression level and poor overall survival; in addition, miR-769-30 tumour expression was identified as an independent prognostic factor (Cox regression analysis). Based on receiver operating characteristic (ROC) curves, serum miR-769-3p level can be utilised to distinguish between healthy individuals and glioma patients [50].

### 2.4. MiRNAs as Therapeutic Targets

To date, temozolomide (TMZ) remains a first-choice chemotherapeutic agent in the treatment of GBMs and astrocytoma. However, patients do not respond to this alkylating agent due to a variety of factors such as MGMT methylation status, drug efflux transporters overexpression and the presence of highly resistant cancer stem cells [78]. Among them, methylation in the promoter of the MGMT gene is one of the most valuable epigenetic markers considering potential treatment response predictors. As MGMT is a DNA repair enzyme, it has an ability to remove methyl residues form nucleotides altered by alkylating drugs. Thus, the hypermethylation of its promoter confers good prognosis in TMZ therapy [5,8]. One direction to fight chemoresistance in cancer is using molecules that impede EMT and EMT-inducing signalling agents, such as those associated with tumour microenvironment. Notably, miRNAs themselves can act as therapeutic molecules due to their tumour suppressive activity [79]. In gliomas, several miRNAs with an impact on classical EMT transcription factors have been implicated as important regulators of TMZ resistance development. For example, miR-181b decreases TMZ resistance by impeding EGFR activity in GBM cells [80]. In turn, miR-128-3p can negatively affect EMT-related proteins, such as c-Met, PDGFRα, Notch1 and Slug, which enhanced glioblastoma TMZ chemosensitivity both in vitro and in vivo [81]. The decreased expression of miR-186-5p contributes to the proliferation and TMZ resistance of GBM cells due to the attenuation of its Twist1-targeting activity [82].

Although many papers point to the therapeutic potential of miRNAs, one particularly interesting study in the field of EMT TF-related molecules in gliomas is by Sadeghipour et al. [51], which aimed to identify a panel of miRNAs targeting different oncogenic pathways in GBMs; these selections could potentially sensitise cancer cells prior to exposure to chemotherapy. A review of the Gene Expression Omnibus (GEO) and The Cancer Genome Atlas (TCGA) databases identified a group of miRNAs and their target genes with essential roles in GBM pathways. Then, different combinations of these miRNAs were studied in GBM cells treated with TMZ or doxorubicin (DOX) by utilising a synthetic miRNA delivery system. Based on cell survival and apoptosis analysis, a panel of five miRNAs consisting of miRNA-138, miRNA-139, miRNA-218, miRNA-490 and miRNA-21 was assessed. The soundness of this particular group was additionally supported by the fact that downstream targets of those rationally selected miRNAs (CDK6, ZEB1, STAT3, TGIF2 and SMAD7) were related to specific, oncogenic pathways (growth and apoptosis, invasion and metastasis, cytokine signalling and stemness). Finally, the increased therapeutic effect of TMZ and DOX was confirmed in vivo, due to this rationally selected miRNAs panel [51]. This approach seems to be particularly relevant, as it reflects the complexity of oncogenic processes and highlights the significance of reciprocal interactions between specific GBM pathways.

## 3. LncRNA Affecting ZEBs

Long non-coding RNAs (lncRNAs) are a functionally diverse group of nucleic acids, which are more than 200 nucleotides in length and generally have no protein-coding potential. To date, a relatively small number of lncRNAs have been annotated with clear functions in the molecular context, despite a multitude of cellular processes which they seem to regulate [83].

Functionally, lncRNAs may affect downstream gene expression via either transcriptional or post-transcriptional regulation. These include transcription interference and chromatin remodelling as well as splicing modifications and post-transcriptional control [84]. There is an abundance of studies in which abnormally expressed lncRNAs have been described as both oncogenic and suppressing molecules in gliomas [85]. Notably, reciprocal interactions between lncRNAs and miRNAs can regulate the expression of many downstream signalling pathways with a great impact on various cellular processes, including glioma progression [86,87]. LncRNAs, which affect cells’ biological functions through ZEB1/ZEB2 signalling in glioma cell lines and tissues, are delineated in Table 2.

Although several mechanisms elucidating the biological role of lncRNAs have been proposed, the most commonly known so far is a competing endogenous RNA (ceRNA) mechanism. CeRNAs (i.e., lncRNAs, some mRNAs and circular RNAs), by binding competitively to certain miRNAs, can impede the regulatory potential which those miRNAs exert on their direct downstream targets (mRNA transcripts), thus modulating gene expression [102,103]. This mechanism is often cited considering functional experiments in gliomas. For instance, in glioma cells lncRNA SNHG5 is bound directly to miR-205-5p, thus preventing the inhibition of its endogenous target, ZEB2 [88]; in another study, lncRNA HOTAIRM1 activated ZEB2 expression by repressing miR-873-5p (a tumour suppressor) [89]. lnRNA UCA1 rescues the inhibitory effect conferred by miR-204-5p on ZEB1 [90]. Cheng et al. [91] described a pathway consisting of lncRNA MALAT1, miR-124 and ZEB2 which contributes to malignant glioma cell proliferation, cell cycle arrest and apoptosis, besides MALAT1 knockdown attenuated tumour growth and had a beneficial impact on the survival in a xenograft mice model.

Notably, some positive feedback loops have been identified among ZEB1-affecting lncRNAs in gliomas. One example is the HOXC-AS2/miR-76-5p/ZEB1 feedback loop. In glioma cells, HOXC-AS2 sponged miR-876-5p to release ZEB1 expression, whereas ZEB1 regulated HOXC-AS2 at the transcriptional level by binding with its promoter [93]. Another study revealed an LINC00511/miR-524-5p/YB1/ZEB1 axis. In GBM cells, lncRNA LINC00511 acted as a ceRNA for miR-524-5p to indirectly upregulate YB1. That promoted ZEB1 expression, which in turn facilitated the transcription of LINC00511. In addition, among a group of 36 GBM tissue samples, a high LINC0051 expression subgroup was positively correlated with a larger tumour size, IDH1/2 wt, recurrence and poor prognosis, as compared with the low expression subgroup. Accordingly, in vivo studies further confirmed the oncogenic potential of this lncRNA [94].

Another interesting pathway including ZEB-related lncRNA concerns the H19/miR-200a/CDK6/ZEB1 axis. Chen and co-workers [41] showed that Cyclin-dependent kinase 6 (CDK6), which is a key component in maintaining the G1 to S phase transition, could promote cell proliferation, and its expression was significantly upregulated in glioma tissue samples and cell lines. Moreover, oncogenic lncRNA H19 was also an inducer of aggressive glioma cell behaviour and targeted miR-200a (which represses oncogenes such as CDK6 and ZEB1) [41]. This conclusion is in line with another study concerning miR-200a activity cited in this review [38].

### 3.1. Hypoxia-Induced EMT

In the context of tumour progression, hypoxia is not only considered a universal characteristic of cancer but also an important modulator of several oncogenic signalling pathways, including those associated with epithelial phenotype [104]. EMT is a complex, biological process that confers epithelial cells with high motility, invasive phenotype and mesenchymal markers expression signature. This promotes extensive infiltration of tumourigenic cells and ultimately leads to the development of distant metastatic lesions [105]. Several reports indicate that hypoxia can induce EMT in some types of human cancer and that the activation of EMT-TFs is prone to hypoxia-associated agents, such as HIF-1α [106,107,108]. Actually, HIF-1 can be considered a master regulator of various oncogenic pathways including TGF-β, Notch and various important transcription factors, whose combined action can promote a dissemination of metastatic cells across a human organism [104].

Zhang et al. [95] showed that lncRNA HOTTIP expression level was positively correlated with HIF-1α in 116 glioma samples and that the high level of HOTTIP was correlated with metastasis when compared with a non-metastatic group (60 tumour tissues with metastasis vs. 56 tumour tissues without metastasis). HOTTIP was significantly upregulated in glioma cell lines treated with hypoxia; additionally, hypoxic conditions promoted mesenchymal marker vimentin as well as aggressive behaviour in examined cell lines. Mechanistically, HOTTIP blocked the expression of mir-101 to upregulate the expression of ZEB1 in glioma cells, thus promoting EMT and metastasis [95]. In another study, Zhao and co-workers proved that hypoxia could induce lncRNA OR7E156P expression in glioma cells [97]. The activity of the OR7E156P/miR-143/HIF-1α axis modulated glioma cell invasion through ZEB1 and HUVEC tube formation. Notably, the authors reported that HIF-1α targeted the promoter region of miR-143, thus repressing its expression. In vivo studies showed that OR7E156P silencing can impede tumour growth in a subcutaneously implanted tumour model in nude mice [97].

To sum up, targeting hypoxic EMT through ncRNAs which interact with master regulators such as HIF1-α should be considered an interesting research direction in unveiling novel therapeutic approaches in the treatment of gliomas.

### 3.2. ZEB1-AS1

To date, lncRNA Zinc Finger E-box-binding homeobox 1 antisense 1 (ZEB1-AS1) has been indicated as an important regulator in many types of tumours, including gastric cancer [109], colorectal cancer [110] and non-small cell lung cancer [111]. In the U7MG glioblastoma cell line, inhibition of ZEB1-AS1 can promote apoptosis and attenuate cell proliferation and invasion [98]. Meng et al. [46] reported that in glioma cells ZEB1-AS1 relieves the repression of ZEB1 caused by two miR-200 family members, miR-200c and miR-141, which are well-known to inhibit the oncogenic functions of both ZEB1 and ZEB2 in gliomas [42,43,44,45]. Additionally, a high level of ZEB1-AS1 expression was correlated with advanced pathological grade and tumour size in a group of 100 primary glioma patients [46]. Lv et al. [99] found that ZEB1-AS1 could affect cell cycle regulation by inhibiting factors such as Cyclin D1 and CDK2 in human glioma cells. That was accompanied by the upregulation of several EMT markers such as ZEB1 and matrix metalloproteinases, including MMP2 and MMP9. Notably, a high expression of ZEB1-AS1 was correlated with poor overall survival and was also closely related to the clinical stage of glioma (82 surgery resection samples) [99]. Likewise, a clinical association analysis by Wei et al. [100] indicated that high ZEB1-AS1 expression in 65 glioma patients was significantly associated with tumour size, KPS and WHO grade. To elucidate aggressive glioma behaviour, the mechanism of ZEB1-AS1-dependent inhibition of miR-577 was proposed. However, the authors did not examine the impact of this axis on the ZEB1 expression level. Notably, both papers reported the role of ZEB1-AS1 as an independent poor prognostic indicator in an examined groups of patients [99,100]. The mechanism by which lncRNA ZEB1-AS1 affects gliomagenesis, by interacting with ZEB1 through miRNAs, remains in the field of interest and undoubtedly requires further research, partially because of its potential prognostic value.

### 3.3. TGF-β

Transforming growth factor β (TGF-β) is an EMT-inducing agent which is essential for cancer progression due to its prominent role in the regulation of cell growth, differentiation and migration. Although TGF-signalling is associated with cancer suppression in the early stages of tumour development, in late-stage tumours it exerts oncogenic function partially through inducing EMT-related factors such as Snail, Slug, ZEB1, ZEB2 and LEF1 [112]. Notably, TGF-β treatment in glioma cells significantly increases the expression of lncRNA UCA1; knockdown of UCA1 decreases Slug expression by releasing the inhibitory activity of miR-1 and miR-203, which are competitively bound by this lncRNA [113]. TGF-β upregulates phospho-SMAD2 (pSMAD2) and ZEB1 expression, thus inducing mesenchymal phenotype in glioblastoma cells [114]. High TGF-β activity predicts poor prognosis in glioma patients [115].

Tang and co-workers [47] found that TGF-β upregulates lncRNA LINC00115 expression level in mesenchymal-like and pro-neural-like glioma stemlike cells (GSCs). LINC00115 was found to bind competitively to miR-200 family (miR-200s) members, miR-200c and miR-200b, thereby releasing ZEB1 from the inhibitory effect of miR-200s. This attenuation of miR-200s by LINC00115 also promoted zinc finger protein 596 (ZNF596) transcription, thus activating ZNF596/EZH2/STAT3 signalling and increasing GSC self-renewal as well as tumour growth in vivo. Finally, LINC00115 expression and correlated co-expression with ZEB1 or ZNF596 were associated with a worse prognosis in GBM patients [47]. Another example of the EMT signalling axis triggered by TGF-β is LINC00645/miR-205-3p/ZEB1. Bioinformatics analysis of GSE490 and TCGA datasets performed by Li et al. [101] showed that LINC00645 expression level was markedly upregulated in glioma samples as compared with normal tissue; additionally, a high level of LINC00645 expression was correlated with poor survival in glioma samples (TCGA and CGGA datasets) as well as in a recruited group of 50 GBM patients. Additionally, LINC00645 has been reported as important regulator of GSC phenotype and impeded tumour growth in vivo.

### 3.4. LncRNA Contributes to EMT-Dependetnt Chemoresistance

Despite continuous advances in the development of novel therapeutic tools, multidrug resistance (MDR) remains a serious issue in the success of cancer therapy [5,116]. In terms of temozolomide (TMZ), a gold standard treatment of GBM, it is well-known that cancer cells ultimately develop resistance to this alkylating agent [78], which may be attributed, at least partially, to the EMT-related signalling pathways. Notably, EMT plays an important role in the development of drug resistance and CSC phenotype in several types of cancer, such as breast and oesophageal cancer [79,117,118]. In gliomas, EMT may significantly affect susceptibility to chemotherapy, as increased ZEB1/ZEB2 and decreased E-cadherin expression levels were observed in TMZ-resistant glioma cells [96]. In the same study, HOTTIP and miR-10b (both oncogenic ncRNAs) were assessed as TMZ resistance-related molecules; expression of lncRNA HOTTIP was especially increased in the particularly resistant cell line. Additionally, miR-10b silencing upon the HOTTIP overexpression promoted TMZ chemosensitivity in all examined glioma cell lines [96]. Another molecule, which may be considered a valuable therapeutic target regarding GBM resistance, is lncRNA MALT1. Li et al. reported that the expression of MALT1 was elevated in two TMZ-resistant cell lines. Furthermore, decreased expression of ZEB1 as well as MDR1, MDR5 and LRP1 (MDR-associated proteins) were observed upon the MALT1 silencing, while its overexpression increased TMZ resistance in a glioma mouse model [92].

### 3.5. LncRNA Controlled by ZEB

Similarly to other EMT transcription factors, it is believed that lnRNAs may be regulated by ZEB family members through the processes of transcriptional repression or activation [102]. In a study by Zhang et al., ZEB1 bound to the lncRNA SBF2-AS1 promoter region with a positive effect on its transcription. Additionally, SBF2-AS1 rescued the inhibition of X-ray repair cross complementing 4 (XRCC4) by sponging miR-151a-3p; as an effect, DNA repair mechanisms intensified. Furthermore, authors reported that TMZ resistance may be acquired in vivo in chemoresponsive GBM areas by SBF2-AS1-enriched exosomes, produced by the intrinsic population of tumourigenic cells [119]. Exosomes are extracellular vesicles that are widespread in the human organism. They range from 50 to 150 nm in diameter and play a primary role in intercellular communication, as they mediate the exchange of various molecules between cells [120]. As exosomes possess potential diagnostic and therapeutic value in tumours [121,122] (serum level of exosomal SBF2-AS1 may be potentially utilised as a TMZ response predictor in GBM patients [119]), further research concerning their function in the pathophysiology of cancer, including gliomas, is still immensely needed.

## 4. Epigenetic Regulation of ZEBs

It is well-known that gliomas can be molecularly characterised considering several epigenetic markers, such as changes in the promoter methylation pattern of MGMT and hTERT genes. Interestingly, IDH1 mutant gliomas (usually associated with secondary gliomas and better prognosis) are closely associated with a CpG island methylator phenotype (G-CIMP) [8]. On the other hand, substantial evidence indicates that EMT signalling is greatly influenced by epigenetic regulation processes, such as chromatin remodelling and histone modifications [123]. Molecular networks of epigenetic reprogramming play an important role in the activation and repression of various EMT-TFs, as well as other associated factors [124,125,126], and affect key molecular pathways associated with hypoxia and stem cell phenotype, thus promoting tumour progression and metastasis [127,128]. For example, histone deacetylase 5 (HDAC5) contributes to the development of chemoresistance in glioma cells [129]. Euchromatic histone-lysine N-methyltransferase 2 inhibitor BIX01294 (Bix) increases the aggressive behaviour of GBM cells by modulating the activity of EMT markers, such as E-cadherin, N-cadherin, β-catenin and Slug [130]. Histone demethylase KDM6B promotes the proliferation, migration and invasion of GBM cells and regulates histone demethylation processes in the Snail promoter [131]. Tumourigenesis and histogenesis of gliomas are modulated by ING5, a tumour suppressor; its overexpression decreases the level of Snail, Slug, Twist1, ZEB1 and ZEB2 in glioma cells and inhibited tumour growth in a xenograft mouse model [132].

Various epigenetic changes in histone methylation patterns may lead to the inhibition of tumour suppressors, but also the repression of various oncogenes, as in the case of the KDM5 subfamily of histone deacetylases [133]. Dai et al. showed that in glioma cells, KDM5A negatively affects cell invasion through H3K4 demethylation-mediated repression of ZEB1; accordingly, bioinformatics analysis of the GSE dataset revealed that metastasis glioma tissue had a lower KDM5A expression level when compared with primary glioma samples. Lower KDM5A levels were associated with poor survival in glioma patients [134].

## 5. Discussion and Future Perspectives

The goal to describe the molecular basis of GBM and other gliomas has been occupying the minds of researchers for decades and is still an ongoing effort. GBM continues to be the most lethal tumour type with still limited treatment options. The intra-tumour heterogeneity, the highly infiltrative nature of lesions and the ability to metastasise, as well as the progressive chemoresistance and blood–brain barrier, contribute significantly to poor outcomes in individuals with GBMs. It has also been proven that due to this genomic complexity, monotherapy will never be effective. Therefore, different therapy approaches are pivotal to increasing treatment response [7]. As pathological EMT is clearly not only an epithelial phenomenon, EMT transcription factors are a good point of interest in gliomas of various grades and have been widely cited as biomarkers and potential therapeutic targets for many years. Among them, ZEB1 and ZEB2 are well-established oncogenes, considering both epithelial and non-epithelial tumours [10,11]. Latest reports showed that the expression level of ZEB1 may be useful as a marker of prognosis in various human cancers [135]. In gliomas, detection of ZEB2 could especially help in assessing patients’ prognosis, which can potentially allow teams to better schedule individual treatment regimens [136].

This review focuses on the microRNA and long non-coding RNA as well as other epigenetic modifications influencing ZEBs in gliomas with the aim of summarising the current state of knowledge regarding their biological functions based on the up-to-date literature. As the above-mentioned ncRNAs seem to have many advantages in terms of potential diagnostic, prognostic and therapeutic utilisation, there is a pressing need to describe them in the context of functional experiments. This approach constitutes a step toward a better understanding of effects set by those ncRNAs on cancer cells, as well as the complex molecular relationships in which they are involved. In addition to ZEBs, there are many other transcription factors with a considerable potential in this regard. As EMT is a complex process which plays a tremendous role in cancer progression and metastasis, many other molecules such as Snail, Slug, Twist1 and Twist2 should be involved in consideration of that matter [10]. Some of them are described widely by various researchers and are considered potentially valuable therapeutic targets. For example, a member of the signal transducer and activator of transcription (STAT) protein family, STAT3 [137], is well-known due to the variety of molecular interactions between a substantial number of ncRNAs and the molecule itself, as well as its direct repressors or activators and other ncRNAs, as comprehensively summarised by Bian et al. [102].

**Figure 1:**
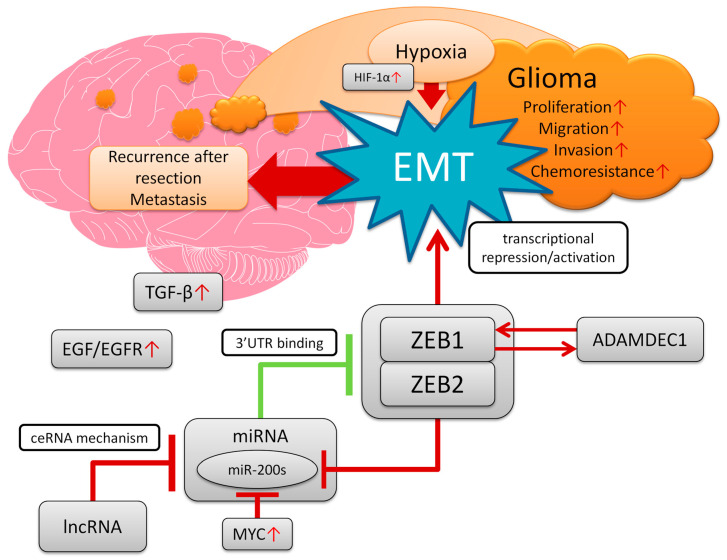
NcRNA with impact on ZEBs in gliomas. Vertical dashes indicate inhibition; arrows—activation; red colour—oncogenic effect, green colour—suppressive effect. Oncogenic LncRNAs, by acting as molecular ‘’sponges’’, impede the inhibitory effect conferred by miRNAs on ZEB1/ZEB2, thus promoting EMT. That confers glioma cells with aggressive, highly motile phenotype, which may promote infiltration to adjacent tissue and intracranial metastases. Note that ADMADEC1 exhibits positive feedback loop with ZEB1 through the miR-203-related axis [48]—that is omitted in the illustration to keep the figure more concise and readable.

MiRNAs are non-coding short RNAs which can act both as suppressors and oncogenes. There is mounting evidence that they affect tumourigenesis in a variety of neoplasms via multiple signalling pathways, including those which are important in tumourigenic EMT. As miRNAs are known to inhibit target mRNAs by binding with their 3′-UTRs, functional experiments considering their effects on oncogenic ZEBs in gliomas are largely focused on those particular ncRNAs that act as tumour suppressors with potential therapeutic value. Consequently, another group of ncRNAs, viz. lncRNAs, are clearly indicated as oncogenes in gliomas based on ZEB functional experiments due to their ability to impede the suppressive action of miRNAs through competing endogenous RNA mechanisms (Figure 1.) A substantial number of reports have investigated SNAI and TWIST, as well as other similar EMT markers. For instance, miR-361-5p inhibits glioma cell migration and invasion and regulates EMT by directly targeting Twist1 [138]. In another study, the lncRNA LINC00152 suppresses miR-107 and promotes N-cadherin, Vimentin and Snail expression [139]. Notably, lncRNA HOTAIRM1 scavenges miR153-5p, a Slug targeting agent. In turn, Slug promotes transcription of HOTAIRM1 in a positive feedback loop [140].

As many miRNAs and lncRNAs are dysregulated in gliomas and can act both as suppressors and oncogenes, it is also of great significance to examine molecules characterised by different mechanisms of action than these canonical ones and determine how they affect ZEBs in glioma tissues and cell lines. It is evident that ncRNAs can also affect EMT-TFs through their upstream regulators and, hence, play both suppressive (lncRNA) and oncogenic (miRNA) roles in gliomagenesis. For instance, lncRNA DGCR5 acts as a tumour suppressor by affecting the DGCR5/miR-21/Smad7 and DGCR5/miR-23a/PTEN axes, and its overexpression upregulates E-cadherin while downregulating Slug and Twist1 in glioma cells [141]. Another option is to pay more attention to those ncRNAs that can be potentially regulated by ZEB1 and/or ZEB2, as ZEBs are commonly known to affect gene transcription in both gliomas and many other tumours.

Although in this work we focused on miRNAs and lncRNAs with impacts on ZEB in gliomas, there are also other groups of ncRNAs which have been reported to regulate transcription factors in human cancer. Notably, among the lncRNAs, circular RNA (circRNAs) are the particular subgroup that is consistently gaining more and more attention in the field [102,103]. To date, they have been indicated as ZEB-related regulators in various malignant lesions, such as breast cancer, non-small cell lung cancer and ovarian cancer [142,143,144]. Although at the time of preparing this manuscript we have not find any reports regarding circRNA with an impact on ZEBs in glioma tissues and cell lines, the subject states another interesting path of research which has already been taken into consideration regarding other canonical EMT transcription factors [145,146].

There is no doubt that in the recent years great progress has been made in the field of molecular basis of glioma. However, the proper designation of particular epigenetic regulators such as ncRNA still remains a big challenge, especially in such molecularly heterogeneous tumours. For instance, to evaluate the particular molecule expression level, researchers have basically three ways by which they can handle that task. They include the utilisation of material derived from clinical samples obtained from a recruited group of patients, retrospective data from bioinformatics tools such as TCGA and glioma/GBM cell lines. Although having different ways to assess expression may strengthen analysis results, each of these ways has its own limitations. Based on the literature reviewed in this work, glioma tissue samples are often derived from a small groups of patients, and those groups usually comprise various types of gliomas, including high grade and low grade or GBM and other gliomas. Usually, the samples are collected from adult groups of patients with primary tumours who have not undergone treatment before resection—although clinically described as low grade or high grade, they lack information regarding their genetic/epigenetic profile, which is of key significance in terms of prognostication and therapy response predictions. Ideally, tissue samples should undergo molecular characterisation, at least in terms of IDH1/2 mut/wt (as in reference [94]). Some of those limitations can be addressed with a proper utilisation of bioinformatics datasets (especially in terms of group size); however, the retrospective character of such tools should be taken into consideration during study design [8]. In terms of established glioma cell lines, it would be interesting to utilise specifically altered cells that could make a sharper focus on a specific type of glioma, such as IDH1/2 mut/wt, drug-specific resistant cells or cells derived from a recruited group of patients, which was actually the case for some studies cited in this review.

## 6. Conclusions

As for now, it is clear that miRNAs and lncRNAs possess significant value in terms of diagnostic, prognostic and therapeutic utilisation in the treatment of cancer. Through their interplay with ZEB1/ZEB2, they impact EMT-related pathways in gliomas, contributing to the proliferation, migration and invasion of tumourigenic cells. That aggressive phenotype may eventually contribute to a tumour recurrence after surgical procedure and the development of chemoresistance. Although in our work we explored various molecular interactions and pathophysiological processes, such as cancer stem cell phenotype, hypoxia-induced EMT and tumour microenvironment, the amount of literature regarding ZEB-related ncRNAs in gliomas is still limited as compared with other human neoplasms. In terms of other epigenetic factors such as histone modification agents, there is even less literature in the field. As ncRNAs are constantly being unveiled regarding their clinical usage in many types of cancer, further progress in the field is warranted, although in the case of gliomas additional obstacles, such as low cerebral bioavailability and the highly heterogenous nature of these tumours, need to be addressed. Ideally, research should have a narrow focus on more specific glioma types with a regard to their genetic and epigenetic characteristics, such as IDH1/2 gene mutations and MGMT methylation status. Considering other TFs with a clear role in EMT-driven gliomagenesis, there is still an urgent need to describe the molecular pathways by which Snail, Slug, Twist1 and Twist2 interact with ncRNAs and other epigenetic factors. Although the existing literature encompasses a range of interesting reports spanning a wide spectrum of topics, such as vasculogenesis, cell differentiation and histone modulators, further progress is necessary to uncover novel biomarkers and potential therapeutic tools.

## Figures and Tables

**Table 1:** MiRNAs with impact on ZEBs in glioma tissue samples/cell lines.

miRNA	Expression in Glioma *	Role in Gliomagenesis	Downstream Targets	Upstream Regulators	Biological Function in Glioma Cells	Reported Prognostic Value **	Refs.
miR-590-3p	Down	Tumour suppressor	ZEB1, ZEB2, N-cadherin, Vimentin, E-cadherin	-	Inhibit migration, invasion	-	[35]
miR-940	Down	Tumour suppressor	ZEB2, N-cadherin, Vimentin, Fibronectin, α-SMA, MMP2, E-cadherin	-	Inhibit migration, invasion	-	[36]
miR-205	Down	Tumour suppressor	ZEB1/Akt/mTOR, E-cadherin, N-cadherin, Vimentin	-	Inhibit migration, invasion	YES	[37,38]
miR-200a	Down	Tumour suppressor	ZEB1/TF	MYC, H19	Inhibit proliferation, migration	-	[39,40,41]
miR-200c	Down	Tumour suppressor	ZEB1/E-cadherin, EGFR, ZEB2	EGFR, MeCP2 /SUV39H1, ZEB1-AS1, LINC00115	Inhibit migration, proliferation, promote apoptosis	-	[42,43,44,45,46,47]
miR-141	Down	Tumour suppressor	ZEB1	ZEB1-AS1	Inhibit migration, proliferation, promote apoptosis	-	[42,46]
miR-203	-	Tumour suppressor	ADAMDEC1	ADAMDEC1/FGF2/FGFR1/ERK1/2/ZEB1	-	-	[48]
miR-622	Down	Tumour suppressor	ZEB2	-	Inhibit proliferation, migration, invasion, promote apoptosis	YES	[49]
miR-769-3p	Down	Tumour suppressor	ZEB2/Wnt /β-catenin	-	Inhibit proliferation, migration, invasion	YES	[50]
miR-139	Down	Tumour suppressor	ZEB1	-	Inhibit migration, invasion	-	[51]

* Expression level in glioma tissue samples/cells as compared with normal brain tissue/non-neoplastic cells; ** based on survival analysis in glioma patients; ‘YES’ indicates that low glioma tissue expression level of particular miRNA can be utilised as an independent poor prognostic indicator and/or is associated with a poor overall survival in glioma patients.

**Table 2:** LncRNAs with impact on ZEBs in glioma tissue samples/cell lines.

lnRNA	Expression in Glioma *	Role in Gliomagenesis	Downstream Targets	Upstream Regulators	Biological Function in Glioma Cells	Reported Prognostic Value **	Refs.
SNHG5	Up	Oncogene	miR-205-5p/ZEB2	-	Promote proliferation	-	[88]
HOTAIRM1	-	Oncogene	miR-873-5p/ZEB2	-	Promote proliferation, inhibit apoptosis	YES	[89]
UCA1	-	Oncogene	miR-204-5p/ZEB1, Fibronectin, COL5 A1	-	Promote migration, invasion	-	[90]
MALAT1	Up	Oncogene	miR-124/ZEB2, ZEB1, MDR1, MRP5, LRP1, E-cadherin, ZO-1, α-SMA, Fibronectin	-	Promote proliferation, inhibit cell cycle arrest, inhibit apoptosis	YES	[91,92]
HOXC-AS2	Up	Oncogene	miR-876-5p/ZEB1, Vimentin, N-cadherin	ZEB1	Promote migration, invasion	YES	[93]
LINC00511	Up	Oncogene	miR-524-5p/YB1/ZEB1, Cyclin D1, CDK4, Vimentin, N-cadherin, E-cadherin	ZEB1	Promote proliferation, migration, invasion, inhibit cell cycle arrest	YES	[94]
H19	Up	Oncogene	miR-200a/CDK6/ZEB1	-	Promote proliferation, invasion, migration	-	[41]
HOTTIP	-	Oncogene	miR-10b/ZEB1/ZEB2 /E-cadherin, VEGF, MMP-9, Vimentin, miR-101/ZEB1	HIF-1α	Promote chemoresistance, proliferation, migration, invasion angiogenesis, metastasis	YES	[95,96]
OR7E156P	Up	Oncogene	miR-143/HIF-1α/ZEB1 /VEGF, Snail, Vimentin	-	Promote invasion, proliferation	YES	[97]
ZEB1-AS1	Up	Oncogene	ZEB1, Snail, MMP2, MMP9, N-cadherin, Integrin-β1, Vimentin, E-cadherin, Cyclin D1, CDK2, Rb, Bax, Bcl-2, miR-200c/miR-141 /ZEB1, miR-577	-	Promote proliferation, invasion, migration, inhibit cell cycle arrest inhibit apoptosis	YES	[46,98,99,100]
LINC00115	Up	Oncogene	miR-200s/ZEB1, Vimentin, E-cadherin miR-200s/ZNF596 /EZH2/STAT3	TGF-β	Promote proliferation	YES	[47]
LINC00645	Up	Oncogene	miR-205-3p/ZEB1, Snail, Vimentin, N-cadherin, E-cadherin, Bcl-2, Bax	TGF-β	Promote proliferation, migration, invasion, inhibit apoptosis	YES	[101]

* Expression level in glioma tissue samples/cells as compared with normal brain tissue/non-neoplastic cells; ** based on survival analysis in glioma patients; ‘YES’ indicates that high glioma tissue expression level of particular lncRNA can be utilised as an independent poor prognostic indicator and/or is associated with a poor overall survival in glioma patients.

## Data Availability

No new data were created or analysed in this study. Data sharing is not applicable to this article.
